# The convenient preparation of stable aryl-coated zerovalent iron nanoparticles

**DOI:** 10.3762/bjnano.6.121

**Published:** 2015-05-21

**Authors:** Olga A Guselnikova, Andrey I Galanov, Anton K Gutakovskii, Pavel S Postnikov

**Affiliations:** 1 Department of Biotechnology and Organic Chemistry, Tomsk Polytechnic University, Tomsk 634050, Russian Federation; 2 Department of General and Inorganic Chemistry, Tomsk Polytechnic University, Tomsk 634050, Russian Federation,; 3 Institute of Semiconductor Physics, Novosibirsk 630090, Russian Federation; 4 Novosibirsk State University, Novosibirsk 630090, Russian Federation

**Keywords:** arenediazonium salts, chemical reduction, covalent modification, surface-modified nanoparticles, zerovalent iron nanoparticles

## Abstract

A novel approach for the in situ synthesis of zerovalent aryl-coated iron nanoparticles (NPs) based on diazonium salt chemistry is proposed. Surface-modified zerovalent iron NPs (ZVI NPs) were prepared by simple chemical reduction of iron(III) chloride aqueous solution followed by in situ modification using water soluble arenediazonium tosylate. The resulting NPs, with average iron core diameter of 21 nm, were coated with a 10 nm thick organic layer to provide long-term protection in air for the highly reactive zerovalent iron core up to 180 °C. The surface-modified iron NPs possess a high grafting density of the aryl group on the NPs surface of 1.23 mmol/g. FTIR spectroscopy, XRD, HRTEM, TGA/DTA, and elemental analysis were performed in order to characterize the resulting material.

## Introduction

Functionalized magnetic nanoparticles (NPs) have aroused great interest recently due to their unique properties and the possibility of widespread applications [1–2]. The modification of magnetic materials may solve a number of high priority problems in medicine and pharmacology [3]. The principal biomedical applications of magnetic NPs include the design of biosensors [4], magnetic resonance imaging [5], targeted drug delivery [6], catalysis [7] and controlled local hyperthermia [8]. Iron is one of the most abundant metals on earth [9]; therefore, the price of precursors for obtaining magnetic iron NPs is inexpensive, thus the modification and synthesis of functionalized iron-containing NPs is a promising area in material science.

There is a great number of well-known modification approaches for the synthesis of functionalized metal NPs [10–11]. One of the principal functionalization methods for metal-containing NPs is based on diazonium salt chemistry [12]. Aromatic diazonium salts (ADSs) are excellent agents for the covalent grafting of organic functional groups onto carbon, metal and metal oxides surfaces [12–13]. Wide applicability of ADSs is due to their ease of preparation, possibility of fast electrolytic reduction, wide range of functional groups of diazonium salt structure, and ability to form stable covalent bonding with surfaces. ADSs have been used to modify metal NPs such as platinum, gold [14], palladium [15], aluminum [16], titanium [17] and iron oxide [18] surfaces. Quite a few works regarding the modification of Fe-based carbon-coated materials using diazonium salts have been published [19–20]. Generally one of the following methods is used to synthesize carbon-coated NPs: reducing flame spray synthesis, flame aerosol synthesis, chemical vapor deposition, etc. However, these procedures require special equipment or techniques [21].

One of the methods for zerovalent iron (ZVI) NP synthesis is chemical reduction. A variety of different approaches have been employed to protect this sensitive material from oxidation, where commonly used methods include coating with carbon [19,22], silica [23], noble metals and oxides [24–26], or the utilization of different surfactants and stabilizers [27]. However, none of these approaches lead to covalent grafting.

In our recent work, the principle of the surface interaction of ZVI NPs and arenediazonium tosylates (ADTs) was demonstrated [28]. However, the mechanism of the modification process and the structure of the surface-modified NPs were not comprehensively investigated. Herein, we report: the detailed study of ZVI NPs with a high grafting density of aryl layers, the structure of the organic coating, the mechanism of the covalent grafting and we emphasize the importance of the obtained material.

## Results and Discussion

The chemical reduction method of ZVI NPs synthesis (reduction of iron salts by sodium borohydride in aqueous or another medium) after isolation of NPs leads to the fast oxidation of powder or passivation by oxides, even at ambient conditions [26–27]. We found that addition of aqueous 4-nitrobenzenediazonium tosylate (ADT) to the freshly prepared aqueous suspension of ZVI NPs (synthesized by reduction of FeCl_3_∙6H_2_O by NaBH_4_ without isolation) leads to the formation of stable functionalized ZVI NPs. The resulting suspension was treated with a magnet for NP precipitates and was subsequently washed 3 times in water, ethanol and acetone. The resulting NP powder was dried in air at 40 °C as previously described [28]. The ADTs were used as a modification agent due to their high storage stability, high stability against explosion and solubility in water, in comparison with classical ADSs [29].

**Figure 1:**
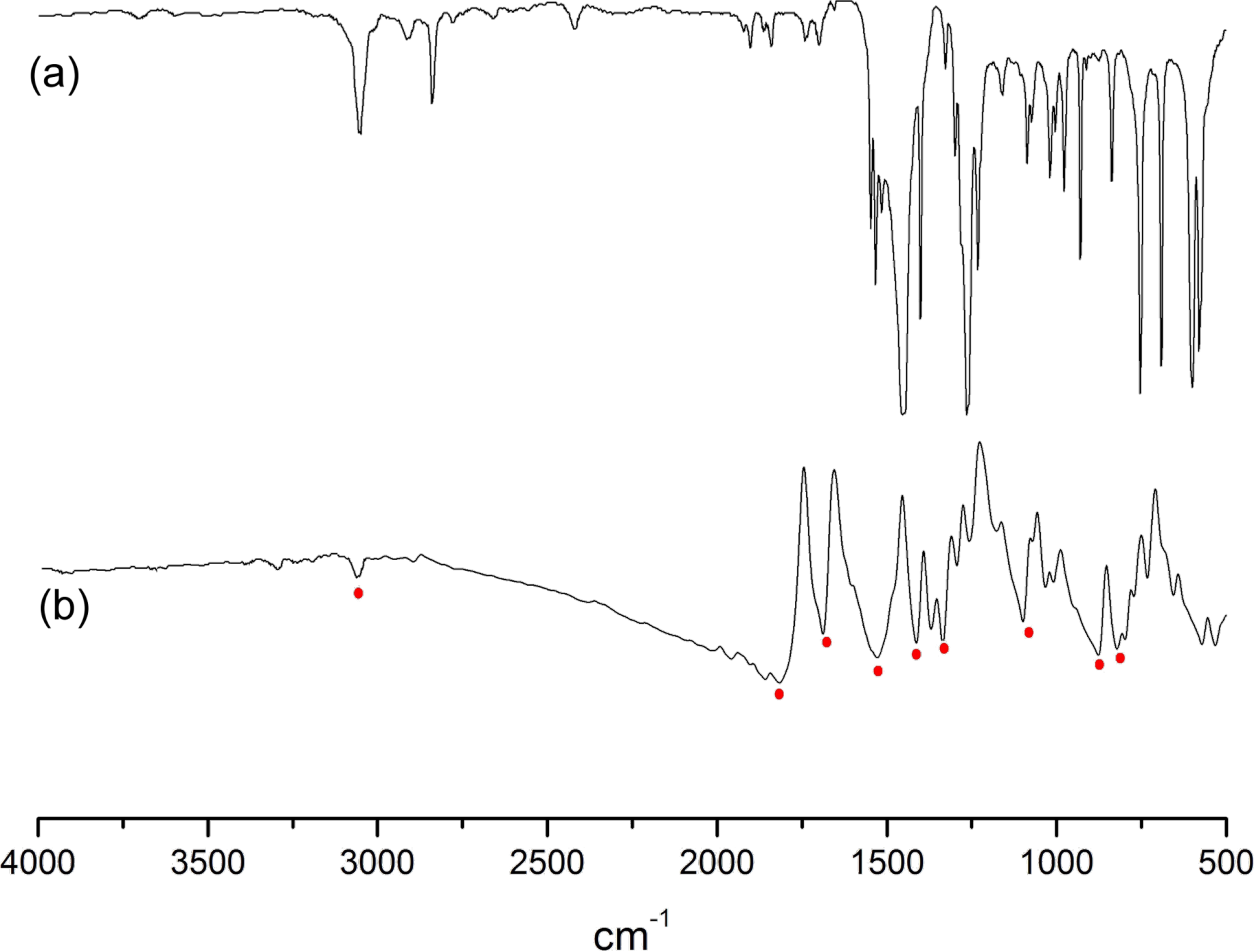
IR spectra of (a) nitrobenzene and (b) ZVI NPs modified by 4-nitrobenzenediazonium tosylate.

IR analysis of the powder in KBr pellets indicates the presence of the 4-nitrophenyl functional group on the NP surface. Figure 1 compares the IR spectrum of nitrobenzene and 4-nitrophenyl-coated ZVI NPs. The NP spectrum exhibits strong adsorption bands corresponding to the benzene ring skeletal vibration in the 1600 cm^−1^ region, while the bending vibration of the C–H bond is associated with the 3050, 1800, 1300–1100 and 800–750 cm^−1^ absorption band regions. This spectrum also contains intense spectral bands at 1550 cm^−1^ and 1350 cm^−1^ related to the vibrations of the nitro group.

To determine the quantity of organic functional groups grafted onto the NPs, thermal gravimetric analysis was carried out in the temperature range 30–600°C with a heating rate of 10 °C/min (see Supporting Information File 1 for details). The modified ZVI NPs showed a weight loss of 14% associated with the decomposition of the aryl layers and evolution of carbon dioxide registered by a mass spectroscopy (MS) detector. The resulting data suggests the formation of a multilayer coating on the NPs surface. The 4-nitrophenyl-coated ZVI NPs exhibited a high thermal stability and did not show any indication of oxidation at temperatures of up to 180 °C.

An additional confirmation of the presence of nitrophenyl groups on the surface of ZVI NPs is obtained from elemental analysis data regarding the content of carbon and nitrogen. Based on this analysis, the ZVI NP powder sample consists of 1.45% N and 9% C, where these values correlate with the composition of the nitrophenyl group. The sample was comprised of 14.95% organic content, which is consistent with 1.23 mmol of nitrophenyl groups/g of NPs (see Supporting Information File 1 for the calculation).

**Figure 2:**
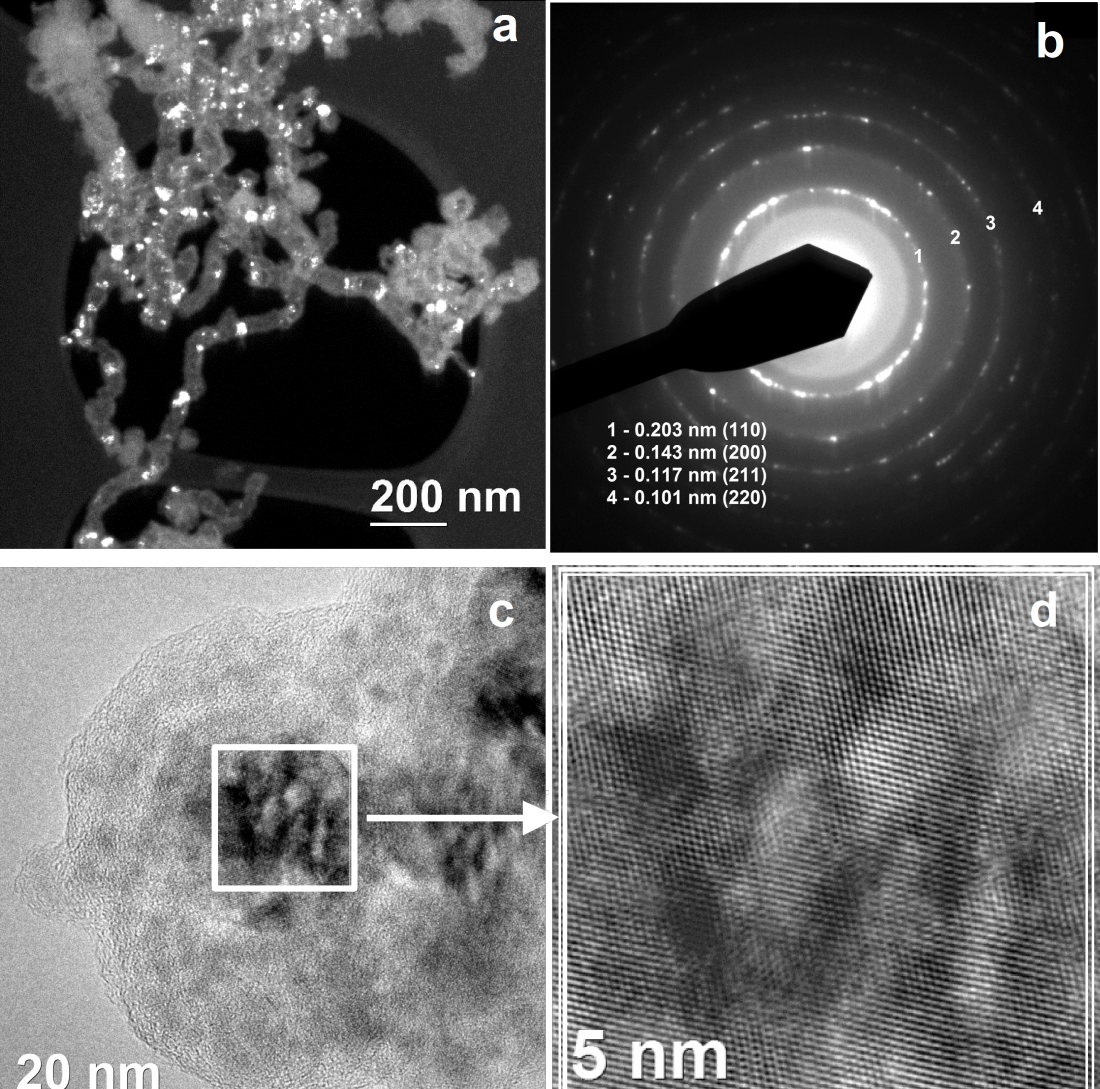
TEM images of 4-nitrophenyl-coated ZVI NPs (a) darkfield image of the NP morphology, (b) FFT image, (c) high-resolution transmission electron (HRTEM) micrograph showing a nearly spherical, crystalline NP with an organic layer coating and (d) HRTEM with high magnification.

TEM analysis revealed nearly spherical particles that were connected in chains. This chain-like morphology is caused by the magnetostatic attraction between the iron particles (Figure 2). Similar observations are also reported in literature [30]. The Fe NPs possess a crystalline structure with space group *Im*3*m* (body-centered cubic lattice with parameter 0.286 nm for α-Fe). In the darkfield image, the nanocrystals are indicated by bright areas. In the high-resolution transmission electron microscopy (HRTEM) image, the atomic planes of the iron crystal lattice are clearly visualized.

The NPs are mostly uniform in size with an average core particle diameter of 21 nm with an organic layer coating of 10 ± 2 nm. The ZVI NP size distribution was calculated by visual particle counting with no less than 500 particles and fitted to a log–normal distribution with a number-based geometric standard deviation of 1.6 according to [22,31]. The mean particle core size was determined to be 21 nm (see Supporting Information File 1). This is the first demonstration of iron-containing NPs modified by ADSs with aryl layers of 10 nm thickness.

**Scheme 1:**
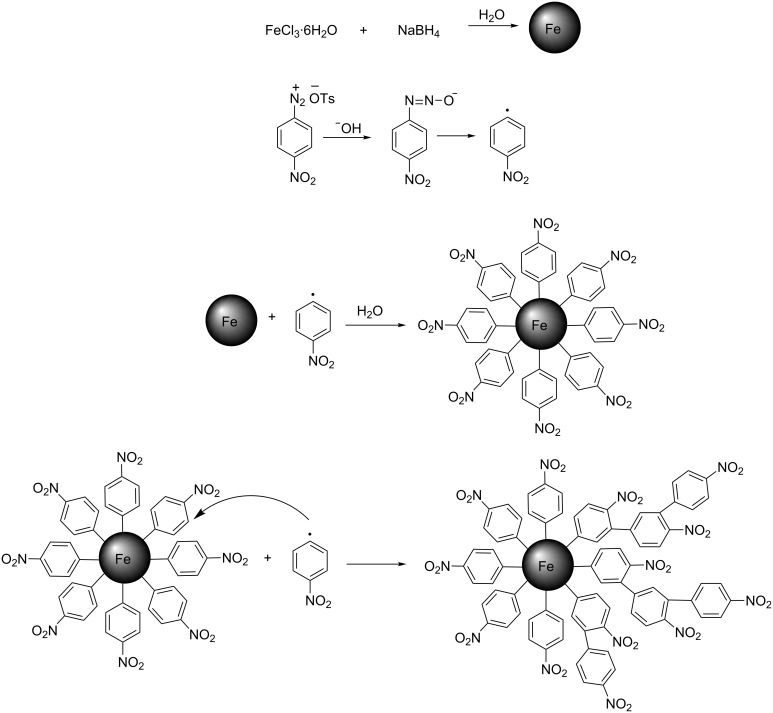
Proposed mechanism of in situ modification of ZVI NPs using ADTs.

We suppose that such a high density of organic functional groups (OFGs) can be explained by our proposed modification mechanism. It was repeatedly shown that diazonium cations give diazoates in basic media. These unstable diazotate species are able to spontaneously generate radicals [18], which react with the surface of the ZVI NPs resulting in highly stable, covalent linkages. Since the first demonstration in 1990, the formation of multilayer films on different surfaces (carbon, gold, iron) has been confirmed several times through the generated radicals from ADSs [32–34]. Since the 4-position group is an electron acceptor, the ortho positions to the substituents are chemically activated and the free radicals can perform a nucleophilic attack. This leads to the well-known aromatic homolytic substitution, S_H_, resulting in multilayer growth as can be seen in Scheme 1. Traces of nitrobenzene, as detected by GC–MS in organic wash solutions after modification, are another confirmation of the radical mechanism of the process. Given these indications of the generation of radicals from ADTs, we hypothesize that there are multilayers of 4-nitrophenyl groups that also contain thick organic layers, as illustrated by TEM.

It is worth mentioning that the characteristics of the NP surface and attached functional groups have a key role for applications [3]. One important characteristic is the content of the functional groups bound to the NP surface [35–36]. Note that using diazonium chemistry, the loading of OFGs on the NP surface depends on the material type and preparation method. Table 1 summarizes the data of the reported metal NPs modified by ADSs and the loading of grafted OFGs.

**Table 1:** Preparation of surface-modified NPs and OFGs loadings.^a^

NP type	modification agent	reaction scheme	loading, mmol/g	ref.

Pt	BF_4_^− +^N_2_C_6_H_4_(CH_2_)_2_CH_3_	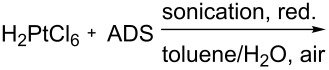	1.5	[14]
Al	BF_4_^− +^N_2_C_6_H_4_CH_2_–DEDTC	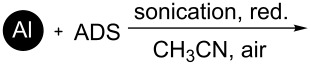	0.46	[16]
Ti	BF_4_^− +^N_2_C_6_H_4_C_6_H_5_	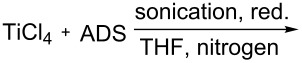	no information	[15]
Pd	BF_4_^− +^N_2_C_6_H_4_C_4_H_9_	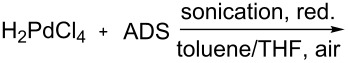	1.12	[15]
Au	AuCl_4_^− +^N_2_C_6_H_4_C_8_F_17_	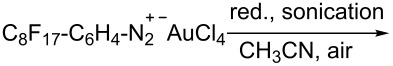	0.48	[37]
Fe_3_O_4_	Cl^− +^N_2_C_6_H_4_NH_2_	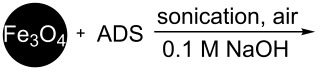	0.024	[18]
Co@C	BF_4_^− +^N_2_C_6_H_4_R, R = NO_2_, –CH_2_–COOH	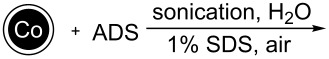	>0.1	[38–39]
Fe_3_C@C	BF_4_^− +^N_2_C_6_H_4_NO_2_	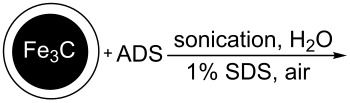	0.3	[19]
Fe, Co, Ni, Ag@C	OTs^− +^N_2_C_6_H_4_R, R = NO_2_, NH_2_, COOH	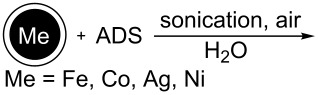	0.12	[20]

^a^red. = reduction agent, DEDTC = *N*,*N*-diethyldithiocarbamate, SDS = sodium dodecyl sulfate.

Table 1 shows that functionalization of metal NPs in basic media leads to the highest loading of OFGs. Nevertheless, a major disadvantage of this method is the utilization of solely lipophilic ADSs in organic solvents (or mixtures with water), which significantly limits applicability of the method. Furthermore, the spontaneous modification of carbon-coated metal NPs results in loadings of less than 0.3 mmol/g. However, magnetic materials with higher grafting density of the aryl or other functional groups could enhance the efficiency of their applications [40].

**Figure 3:**
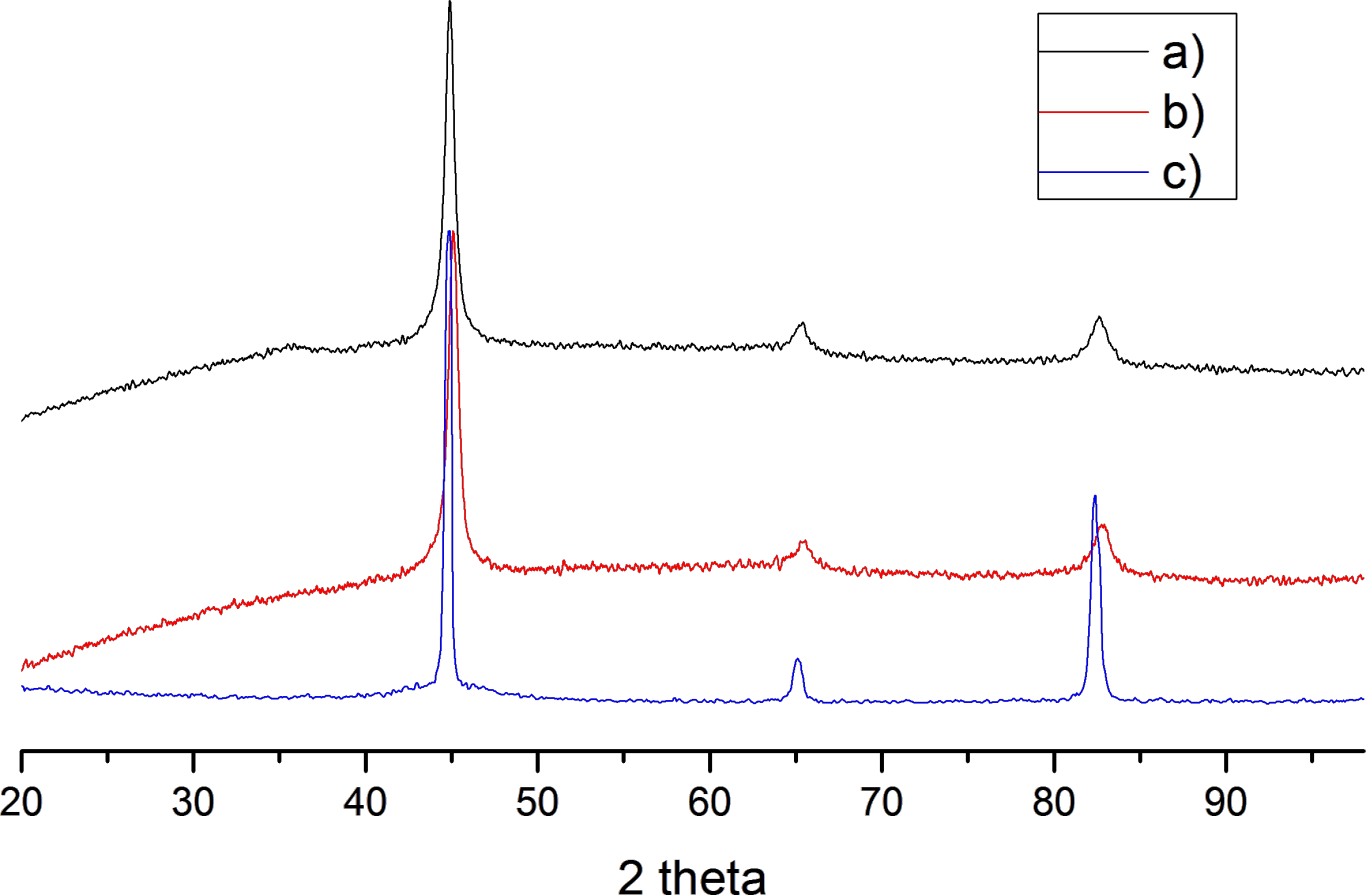
XRD pattern of (a) freshly prepared 4-nitrophenyl-coated ZVI NPs, (b) corresponding particles after 6 months, and (c) bcc α-Fe (JCPDS File # 06-0696).

XRD analysis was applied to characterize the freshly prepared sample of modified ZVI NPs and the sample again after storage in air for at least 6 months (Figure 3). It is worth noting that there were no significant differences in the XRD patterns between two samples, which is explained by the protective function of the aryl layer coating. XRD patterns of both samples showed only the characteristic peaks of ZVI at 2θ = 45, 65, and 83°. Afterwards, a qualitative composition of the samples was determined: both modified NP powders were composed of 100% ZVI iron. We conclude that the aryl layer on the NP surface was able to prevent the oxidation process.

## Conclusion

In summary, we have reported the detailed study of aryl-coated ZVI NPs, synthesized by the room temperature reduction of iron chloride in an aqueous medium using ADTs. The resulting NPs with an average particle core diameter of approximately 21 nm maintain high oxidation resistance and air stability for at least 6 months. The high-density, 10 nm aryl multilayer (loading of OFGs is 1.23 mmol/g) should provide enhanced biomedical properties. The developed strategy allows for the efficient and easy synthesis of pure iron-functionalized NPs. Methods such as FTIR, XRD, HREM, TGA/DTA, and elemental analysis were used to characterize the structure of the obtained ZVI NPs. We also believe that this novel synthetic approach will not only be useful for the preparation of iron NPs but also provide a general functionalization strategy for pure metal NPs. We also conclude that this approach can be applicable for medicinal research since water is the reaction solvent and it produces materials with a high anticipated magnetic moment, stable covalent binding, and a high density of functional groups. The proposed synthesis strategy of ZVI NPs with a wide range of attached organic functional groups allows for the design of contrast agents for theranostic applications.

## Experimental

### Materials and instrumentation

*tert*-Butyl nitrite, 4-toluenesulfonic acid (p-TsOH), 4-nitroaniline, iron trichloride hexahydrate (FeCl_3_∙6H_2_O), and sodium borohydride were purchased from Aldrich and were used without further purification. Deionized water was used for all chemical reactions. All the solvents were obtained from Aldrich and used as received.

Powder X-ray diffraction data were collected on a Shimadzu XRD-7000 diffractometer (30 min) using Cu Kα radiation (1.5405 Å). The FTIR spectra were measured using the KBr pellet technique with a Nicolet 5700 spectrometer ranging from 400 to 4000 cm^−1^. Differential thermal and thermogravimetric analysis was carried out on a SDT Q60 thermal analyzer in the temperature range 20–600 °C with a heating rate of 10 °C/min under a flow of air at 80 mL/min. HRTEM observations were performed on a JEM-4000EX (JEOL) electron microscope. Elemental analysis was acquired with a Leco 628 carbon/hydrogen/nitrogen analyzer. The specific surface area was measured using a Micromeritics Tristar II 3020 surface area analyzer and was calculated from the isotherm of low-temperature nitrogen adsorption. The GC-MS were recorded using an Agilent 7890A GC device with a mass selective detector (Agilent 5975C) and helium as a carrier gas. The magnetization curves of the surface-modified ZVI NPs were investigated in pulsed magnetic fields up to 35 T at a temperature of 77 K with a SQUID magnetometer in magnetic fields up to 15 kOe at 300 and 2 K.

### Synthesis of 4-nitrobenzenediazonium tosylate

To a solution of p-TsOH (1.425 g, 7.5 mmol) in acetic acid (12 mL), *tert*-butyl nitrite was slowly added (0.9 mL, 7.5 mmol). Next, 4-nitroaniline (0.7 g, 5 mmol) was added in 4 steps to the reaction mixture over 1 min. The mixture was stirred for 30–40 min until TLC indicated the complete consumption of the amine (hexane/ether 1:1). After completion, the reaction mixture was precipitated by adding diethyl ether (200 mL). The precipitate was washed with diethyl ether, filtered under reduced pressure and dried under vacuum.

Caution! Diazonium salts in the dry state are potentially explosive. Therefore, they must be carefully stored and handled.

Mp 132 °C; IR (KBr): 2308 (N≡N); ^1^H NMR (300 MHz, DMSO); δ 2.28 (s, 3H), 7.10 (d, *J* = 7.5 Hz, 2H), 7.47 (d, *J* = 7.5 Hz, 2H), 8.69 (d, *J* = 9.3 Hz, 2H), 8.89 (d, *J* = 9.3 Hz, 2H); ^13^C NMR (75 MHz, DMSO); δ 20.84, 121.96, 125.57, 126.04, 128.18, 134.60, 137.85, 145.55, 153.22.

### Synthesis of 4-nitrophenyl-coated ZVI NPs

The synthesis of ZVI NPs was based on the work presented in [41]. An aqueous solution (15 mL) of FeCl_3_∙6H_2_O (0.406 g, 1.5 mmol) was slowly added to an aqueous solution (10 mL) of NaBH_4_ (0.171 g, 4.5 mmol) under vigorous mechanical stirring. The color of the solution changed immediately from yellow to dark, indicating the formation of nanoparticles. After 10 min, the aqueous solution (15 mL) of 4-nitrobenzenediazonium tosylate (0.68 g, 2.25 mmol), as synthesized above, was added directly to the reaction vessel. The mixture was stirred for 30 min, and completion of the reaction was detected via conversion of 4-nitrobenzenediazonium tosylate by the test for β-naphthol. The obtained suspension was washed by 9 cycles of magnetic separation/redispersion in water (3×), ethanol (3×) and acetone (3×) and dried at 40 °C.

## Supporting Information

File 1 Additional experimental data and calculations. Functional group calculation by elemental analysis, TGA/DTA curves, core size distribution, specific surface area and magnetization curve.
