# Supplementary material for: The convenient preparation of stable aryl-coated zerovalent iron nanoparticles
Source: Beilstein J Nanotechnol. 2015 May 21;6:1192–8. doi: 10.3762/bjnano.6.121 (PMC4464190; doi:10.3762/bjnano.6.121)
Supplement: File 1 — Additional experimental data and calculations. Functional group calculation by elemental analysis, TGA/DTA curves, core size distribution, specific surface area and magnetization curve. [file Beilstein_J_Nanotechnol-06-1192-s001.pdf]

Supporting Information

for

The convenient preparation of stable aryl-coated zerovalent iron nanoparticles

Olga A. Guselnikova[1], Andrey I. Galanov[2], Anton K. Gutakovskii[3,4] and Pavel S. Postnikov[2]*

Address: [1]Department of Biotechnology and Organic Chemistry, Tomsk Polytechnic University, Tomsk 634050, Russian Federation, [2]Department of General and Inorganic Chemistry, Tomsk Polytechnic University, Tomsk 634050, Russian Federation, [3]Institute of Semiconductor Physics, Novosibirsk 630090, Russian Federation, and [4]Novosibirsk State University, Novosibirsk 630090, Russian Federation

Email: Pavel S. Postnikov* - postnikov@tpu.ru

* Corresponding author

Additional Experimental Data and Calculations

Calculation of functional group by elemental analysis

According to elemental analysis, the NPs consist of 9% carbon and 1.45% nitrogen, which closely corresponds to the composition of the nitrophenyl group. By the ratio calculation the sample consists of 4% oxygen and 0.5% hydrogen. Therefore, the sample consist of 85.05% iron and 14.95% organic layer. Finally, the nitrophenyl group has a molar weight of 122 g/mol, and 14.95% is correlated to 1.23 mmol/g of NPs.

TGA/DTA analysis

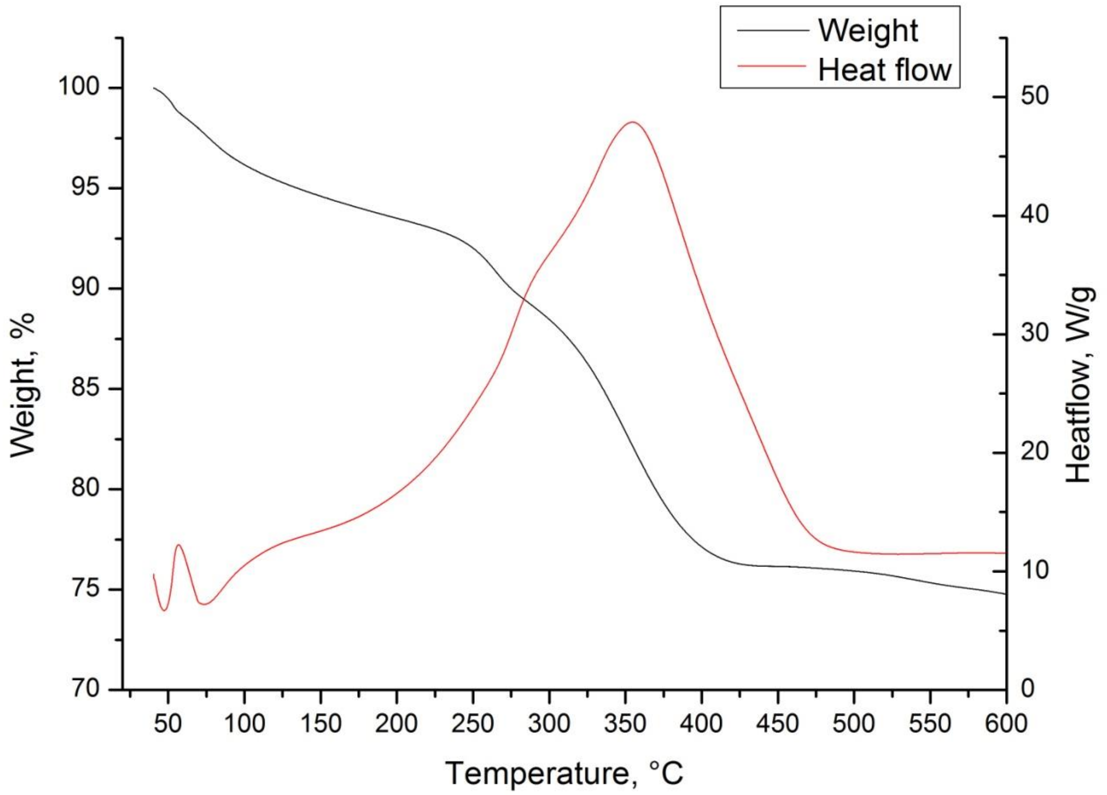

Figure 1: TGA/DTA analysis of ZVI NPs coated with 4-nitrophenyl groups.

Thermal analysis was carried out in order to observe thermal behavior of modified NPs and quantify the organic coating of the produced NPs in the temperature range of 30–600°C with a heating rate of 10°C/min. In the temperature range of 30–200 °C the low weight loss was assumed to be due to the desorption of water or some other adsorbed gases. A total weight loss of organic functional groups is 13% was observed between 200 and 450 °C and accompanied with exothermal effect as seen in Figure 1.

TEM image and size distribution of produced NPs

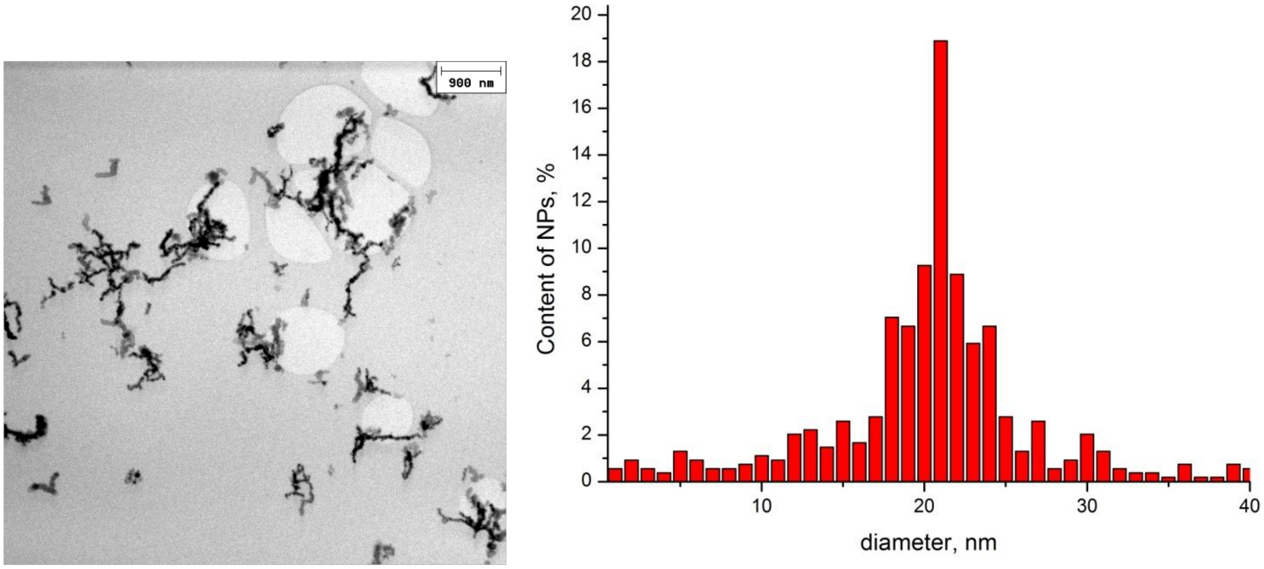

Figure 2: Overview of 4-nitrophenyl-modified ZVI nanoparticles (left), core size distribution (right).

The ZVI particle cores size distribution was evaluated by visual particle counting of at least 500 particles and fitted to a log-normal distribution with a number-based geometric standard deviation of 1.6 and a mean particle core size of about 21 nm.

Specific surface area

The specific surface area was determined by low-temperature nitrogen adsorption. The BET surface area is 92.7 m^2/g and the pore diameter size is 1.72 nm, which corresponds to low-porous near the spherical nanocrystals.

Magnetization curve

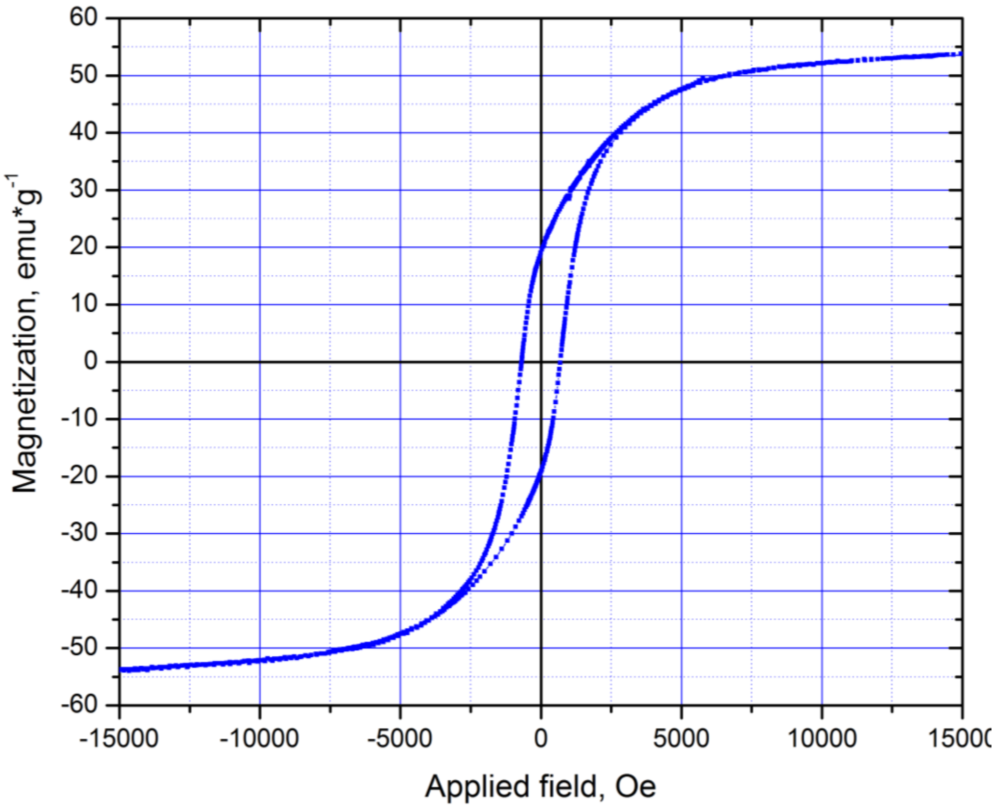

Figure 3: Hysteresis loops for surface-modified ZVI NPs.

The aryl-coated NPs saturation magnetization is 55 emu·g^{-1}.
